# QTL underlying resistance to two HG types of Heterodera glycines found in soybean cultivar 'L-10'

**DOI:** 10.1186/1471-2164-12-233

**Published:** 2011-05-12

**Authors:** Wei Chang, Limin Dong, Zizhen Wang, Haibo Hu, Yingpeng Han, Weili Teng, Hongxia Zhang, Maozu Guo, Wenbin Li

**Affiliations:** 1 Soybean Research Institute (Key Laboratory of Soybean Biology of Chinese Education Ministry), Northeast Agricultural University, Harbin, 150030, China; 2 National Key Laboratory of Plant Molecular Genetics, Shanghai Institute of Plant Physiology and Ecology, Chinese Academy of Sciences, Shanghai, 200032, China; 3 Department of Computer Science and Technology, Harbin Institute of Technology, Harbin, 150001, China

## Abstract

**Background:**

Resistance of soybean (*Glycine max *L. Merr.) cultivars to populations of cyst nematode (SCN; *Heterodera glycines *I.) was complicated by the diversity of HG Types (biotypes), the multigenic nature of resistance and the temperature dependence of resistance to biotypes. The objective here was to identify QTL for broad-spectrum resistance to SCN and examine the transcript abundances of some genes within the QTL.

**Results:**

A Total of 140 F_5 _derived F_7 _recombinant inbred lines (RILs) were advanced by single-seed-descent from a cross between 'L-10' (a soybean cultivar broadly resistant to SCN) and 'Heinong 37' (a SCN susceptible cultivar). Associated QTL were identified by WinQTL2.1. QTL Qscn3-1 on linkage group (LG) E, Qscn3-2 on LG G, Qscn3-3 on LG J and Qscn14-1 on LG O were associated with SCN resistance in both year data (2007 and 2008). Qscn14-2 on LG O was identified to be associated with SCN resistance in 2007. Qscn14-3 on LG D2 was identified to be associated with SCN resistance in 2008. Qscn14-4 on LG J was identified to be associated with SCN resistance in 2008. The Qscn3-2 on LG G was linked to Satt309 (less than 4 cM), and explained 19.7% and 23.4% of the phenotypic variation in 2007 and 2008 respectively. Qscn3-3 was less than 5 cM from Satt244 on LG J, and explained 19.3% and 17.95% of the phenotypic variations in 2007 and 2008 respectively. Qscn14-4 could explain 12.6% of the phenotypic variation for the SCN race 14 resistance in 2008 and was located in the same region as Qscn3-3. The total phenotypic variation explained by Qscn3-2 and Qscn3-3 together was 39.0% and 41.3% in 2007 and 2008, respectively. Further, the flanking markers Satt275, Satt309, Sat_350 and Satt244 were used for the selection of resistant lines to SCN race 3, and the accuracy of selection was about 73% in this RIL population. Four genes in the predicted resistance gene cluster of LG J (chromosome 16) were successfully cloned by RT-PCR. The transcript encoded by the gene Glyma16g30760.1 was abundant in the SCN resistant cultivar 'L-10' but absent in susceptible cultivar 'Heinong 37'. Further, the abundance was higher in root than in leaf for 'L-10'. Therefore, the gene was a strong candidate to underlie part of the resistance to SCN.

**Conclusions:**

Satt275, Satt309, Sat_305 and Satt244, which were tightly linked to the major QTL for resistance to SCN on LG G and J, would be candidates for marker-assisted selection of lines resistant to the SCN race 3. Among the six RLK genes, Glyma16g30760.1 was found to accumulate transcripts in the SCN resistance cultivar 'L-10' but not in 'Heinong 37'. The transcript abundance was higher in root than in leaf for L-10.

## Background

Soybean (*Glycine max *(L.) Merr.) is the most important economic and nutritional legume crop for both oil and protein products worldwide [1]. However, a variety of abiotic stresses and biotic stress are threatening the world's soybean production every year. After scarcity of water, seed yield losses in soybean are mainly due to pest and pathogen infections [2]. One of the most destructive pests of soybean is the soybean cyst nematode (SCN; *Heterodera glycines *Ichinohe). It can cause various symptoms after infection such as chlorotic patches within leaflets, root necrosis and suppression of root growth. Yield losses in the world due to SCN infection have approached $2 billion a year [3]. Once established in a field, the infestations have been difficult to eradicate. Eight races of SCN are found (1, 2, 3, 5, 6, 7, 9 and 14) [4] in China. Race 3 is predominant in the Northeastern Provinces of China (including Heilongjiang, Jilin, Liaoning and Inner Mongolia).

Although biological controls and transgenic technology have some promise, breeding the pest-resistant materials was still the only effective control method by 2010 [5]. There were more than 100 soybean accessions from Asia that contained SCN resistance genes, but only a few of them had been utilized for developing US cultivars and nearly all the resistance genes in developed varieties were from two sources, 'Peking' and 'PI88788' [6].

Inheritance of resistance to SCN was first reported in Peking when three resistance genes (*rhg1,rhg2 and rhg3*) were identified [7]. The *rhg1 *locus has been shown to have the greatest impact on the development of SCN from all HG Types in several resistance sources including Peking, PI437654 and PI88788. Although originally reported as a recessive locus, the *rhg1 *locus has more recently been characterized as incomplete dominant or dominant depending on the HG Type 0 used [8]. The fourth resistance gene *Rhg4 *had been confirmed as being necessary for full resistance to some races of SCN in Peking but not PI88788. It behaved as a dominant gene in Peking-derived sources. Both *Rhg1 *and *Rhg4 *were necessary for the full resistance to SCN race 3 and 14. An additional dominant gene was identified in PI88788 and was designated *Rhg5 *that also had been characterized as a dominant gene [9].

HG Types and plant resistance were both shown to be temperature dependent. As the traditional breeding process is time consuming and labor intensive, new cultivar breeding usually could not adapt with the speed of drift among pathogen populations driven by mutations within massive field populations. Therefore, new methodologies for SCN resistance breeding were required. Genetic marker technology has facilitated the identification, localization and characterization of QTL associated with important agronomic traits including SCN resistance [7]. DNA-based techniques such as micro satellites (SSR) have been used extensively for soybean gene mapping, because they are abundant, uniformly distributed, highly polymorphic, codominant, rapidly produced by PCR, relatively simple to interpret, and easily accessed by other laboratories via published primer sequences. Further, they have been used to anchor the soybean genome sequence [10]. Consequently, numerous publications have appeared on the identification and localization of QTL underlying resistance to SCN [7]. The marker assisted selection provided the potential to develop SCN resistance in soybean cultivars tested in single environments, often in greenhouse assays. For instance, Soybean JTN-5503, JTN-5303 (high yield, with resistance to multiple nematode populations), were the first soybean lines developed using MAS for nematode resistance [11,12].

The SCN resistance gene *Rhg4 *was inferred to be linked with pBLT24, pBLT56 and *I *gene for seed coat color on linkage group A2 (chromosome 8) [13]. QTL identified and localized for SCN resistance based on both genotypic and phenotypic data in a segregating population was first reported by Concibido et al. (1994)[14]. During the following decade, a total of 60 SCN resistant QTL were reported on the following linkage groups: A1, A2, B1, B2, C1, C2, D1a, D2, E, F, G, H, I, J, L, M, and N [7]. QTL on the LG G near *rhg1 *was detected in most studies and was considered to be one of the major genes underlying resistance to SCN. Nearly 80% of these studies used the SCN HG Type 0 (race 3) resistance because it was the main cause of yield reduction among soybeans grown in many areas, including Northeast of China. A minor QTL S16-5 on LG K provided 5% additive effects for resistance to SCN race 1 had been reported in 2009, this was the first report of a QTL on linkage group K associated with SCN resistance [15]. Another QTL was detected on LG D2 (Satt574; P = 0.001, R2 = 11%) and associated with the resistance to HG Types 1.3- and 1.2.5- [6].

Candidate genes underlying QTL were identified and one, a lecine-rich repeat (LRR) trans-membrane (TM) receptor like kinase (RLK) was tested in transgenic plants [8]. Resistance was provided to SCN and a fungal pathogen, *Fusarium virguliforme*. In contrast, another RLK in the *Rhg4 *interval was shown not to underlie the resistance locus [16]. Candidate genes underlying other loci were not reported to date.

Cultivars with broad-spectrum resistance to SCN were available in China and may be more useful than those that had specific resistance. 'Heinong 37' was shown to be genetically distinct from SCN resistant cultivars with high yielding under continuous culture [17]. 'L-10' was resistant to different races of SCN (by our unpublished data). The objectives of the present study were to confirm the previously reported QTL, to identify new QTL associated with broad-spectrum resistance to multiple SCN races and to examine candidate genes underlying selected QTL using the cross of 'Heinong 37' and 'L-10'.

## Results

### Linkage Analysis

A total of 711 SSR markers were used for detecting polymorphisms between the two parents, and 211 of them (29.7%) were polymorphic among the RILs. It was remarkable that the ratio of polymorphic markers was extremely high on LG G and LG J (about 53% and 50%). A total of 151 SSR markers that were polymorphic among RILs were mapped onto the integrated soybean linkage map designed by Song et al. [18]. Chi-square tests of these markers fitted well the expected ratio of 1:1 for genotypes AA: BB.

### Female index analysis based on the greenhouse data

**Figure 1:**
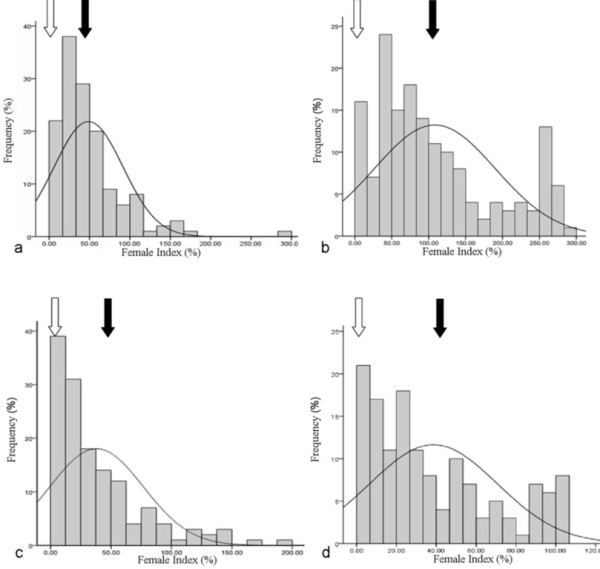
**Distribution of Female Index among 140 RILs**. Filled arrows represent mean values for the RILs, empty arrows respresent mean values for 'L-10'. a: SCN race 3 in 2007, b: SCN race 3 in 2008, c: SCN race 14 in 2007, d: SCN race 14 in 2008.

The female index was significantly different between the two parents, 'L-10' (mean 0.8%) and 'Heinong 37' (mean 79.9%). The mean female index across the RIL population was 48.8 to 109.2 for race 3 and 38.1 to 43.3% for race 14 in 2007 and 2008, respectively. The range of female index for the race 3 was much wider than that for the race 14 in both 2007 and 2008 (Table 1). The Shaprio-Wilks tests showed that the frequency distribution of female index significantly deviated from normal distribution model (W = 0.830, P < 0.0001 for race 3 in 2007; W = 0.822, P < 0.0001 for race 14 in 2007; W = 0.891, P < 0.0001 for race 3 in 2008; W = 0.80, P < 0.0001 race 14 in 2008). However, using the SAS normal logarithm conversion of the data resulted in normal distributions for these female index data (race 3 in 2007: W = 0.92, P = 0. 2274; race 14 in 2007: W = 0.91, P = 0.1897; race 3 in 2008: W = 0.92, P = 0.3544; race 14 in 2008: W = 0.94, P = 0.2679) (Figure 1).

**Table 1:** The mean percentage of SCN female index for parents and RILs inoculated with different SCN races in the greenhouse

SCN races	FI of 'L-10'	FI of 'Heinong 37'	Average FI in RILs	The FI Range in RILs
07-03^a^	0.6	56.4	48.8	0-291.0
07-14^b^	1.6	80.3	38.1	0-190.9
08-03 ^c^	0.0	94.3	109.2	0-284.0
08-14 ^d^	1.1	88.6	43.4	0-106.3

^a ^07-03: RILs inoculated with the SCN race 3 in 2007; ^b ^07-14: RILs inoculated with the SCN race 14 in 2007; ^c ^08-03: RILs inoculated with the SCN race 3 in 2008; ^d ^07-03: RILs inoculated with the SCN race 14 in 2008; FI = (Number of cysts and females on detected plant)/(Average number of cysts and females on 'Lee68') × 100.

### QTL analyses based on the greenhouse loss data

**Figure 2:**
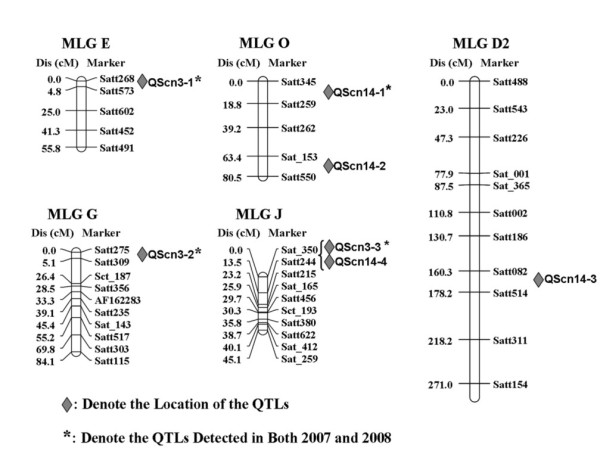
**Genomic locations of identified QTL affecting SCN resistance**. Genomic locations of identified QTL affecting SCN resistance of the F_7 _RIL population of 'L-10' and 'Heinong 37' based on the greenhouse female index for race 3 and race 14 respectively in 2007 and 2008. The map distances in cMs were shown on the left. The QTL locations were indicated on the right.

The map distance between molecular markers and the associated QTL were calculated by WinQTL 2.1. QTL were relevant to SCN resistance based on the greenhouse female index data in this study (Figure 2). QTL Qscn3-1 on MLG E was detected in the interval Satt573-Satt268 that explained 11.36% and 7.14% of the phenotypic variation for SCN race 3 resistance in 2007 and 2008. A major QTL, Qscn3-2, was located on MLG G in the interval Satt275-Satt309 and accounted for 19.73% and 23.36% of the phenotypic variation for SCN race 3 resistance in 2007 and 2008, respectively. On MLG J, Qscn3-3 was detected in the interval Sat_350-Satt244 that explained 19.3% and 17.95 of the phenotypic variation for SCN race 3 resistance in 2007 and 2008, respectively. In the same interval, QTL Qscn14-4 was mapped approximately 3.3 cM from the marker Satt244 and accounted for 12.62% of the phenotypic variation for SCN race 14 resistance in 2008. There were two QTL-Qscn14-1 and Qscn14-2 for SCN race 14 resistance were detected on MLG O in the intervals Satt345-Satt259 and Satt153-Satt550 that explained 8.84% and 4.97% of the phenotypic variance in both years, and 15.14% in 2007. QTL Qscn14-3 accounting for 6.8% of the phenotypic variation was found on MLG D2 between Satt082 and Satt514 for SCN race 14 resistance in 2008 (Table 2).

**Table 2:** Markers associated with SCN resistance based on the greenhouse female index data

QTL	MLG	Years	Interval	Marker	Dis (cM)	**R**^**2 **^**(%)**^**a**^	**LOD **^**b**^	**Allelic means ± SEM**^**c**^	
								
								L-10	Heinong37
Qscn3-1	E	2007	Satt573-Satt268	Satt573	2.65	11.36	3.0788	15.35 ± 6.32	65.56 ± 9.22
Qscn3-1	E	2008	Satt573-Satt268	Satt573	2.26	7.14	2.0428	19.21 ± 10.22	86.36 ± 9.26
Qscn3-2	G	2007	Satt275-Satt309	Satt309	3.14	19.73	3.3728	10.21 ± 5.27	68.44 ± 8.12
Qscn3-2	G	2008	Satt275-Satt309	Satt309	3.92	23.36	2.3952	12.23 ± 5.42	90.12 ± 10.39
Qscn3-3	J	2007	Sat_350-Satt244	Satt244	4.3	19.3	2.5631	19.36 ± 12.36	63.92 ± 5.97
Qscn3-3	J	2008	Sat_350-Satt244	Satt244	2.6	17.95	3.0245	19.65 ± 5.36	85.17 ± 11.45
Qscn14-1	O	2007	Satt345-Satt259	Satt345	9.96	8.84	2.5374	24.97 ± 7.09	60.67 ± 9.19
Qscn14-1	O	2008	Satt345-Satt259	Satt346	7.39	4.97	2.2664	21.17 ± 15.32	88.6 ± 12.69
Qscn14-2	O	2007	Satt153-Satt550	Satt550	11.27	15.14	4.1175	19.32 ± 11.51	70.83 ± 15.78
Qscn14-3	D2	2008	Satt082-Satt514	Satt082	1.27	6.8	2.4523	16.39 ± 6.69	73.92 ± 11.58
Qscn14-4	J	2008	Sat_350-Satt244	Satt244	3.3	12.62	2.0563	17.63 ± 9.18	68.29 ± 10.63

^a ^R^2 ^was the proportion of the phenotypic data explained by the marker locus; ^b ^LOD was log of odd score; ^c ^SEM (standard error mean): SD√N where SD was standard deviation and N was the number of each of allele.

The flanking markers Sat_350 and Satt244 could be used for the selection of the QTL that underlay both resistances to SCN race 3 and SCN race 14. The total phenotypic variation explained by Qscn3-2 and Qscn3-3 together was 39.0% and 41.3% for the year 2007 and 2008, respectively. Further, the flanking markers Satt275, Satt309, Sat_350 and Satt244 were used for the selection of resistant lines to SCN race 3. The accuracy of selection was about 73% in this RILs population from the cross between 'L-10' and 'Heinong 37' (data not shown).

### Candidate gene detection and transcript abundance analysis

**Figure 3:**
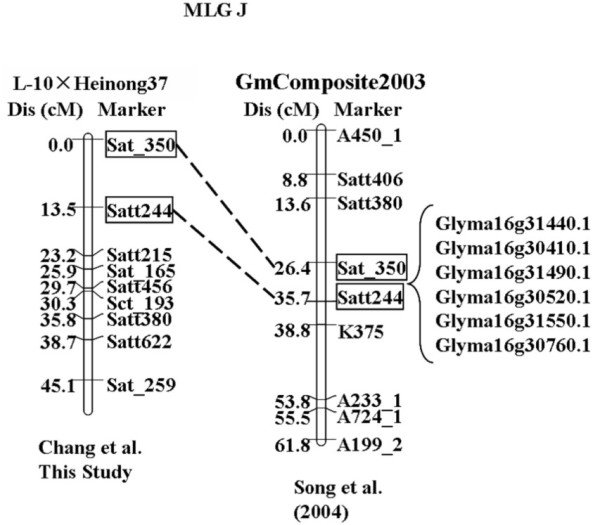
**Projecting and genetic map integration of MLG J**. The markers framed were common markers of these two maps. The map distances in cMs were shown on the left. The QTL locations were indicated on the right.

**Figure 4:**
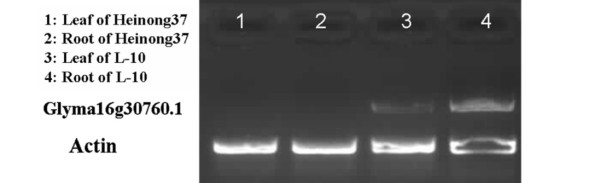
**Result of semi-quantitative RT-PCR for Glyma16g30760.1 transcript abundance**. Actin: an internal standard β-actin.

All the QTL mentioned above were projected on the genetic map GmComposite2003 based on the homothetic function. For the interval between Sat_350 and Satt244 that contained the genes underlying resistance to both SCN race 3 and SCN race 14, the QTL was first projected on the genome map according to the physical distance ratio of genetic distance of 412 kbp/cM between the two DNA markers. A total of 343 putatively gene encoded sequences encompassed in this region were used for resistance gene analog (RGA) detection by the hmmsearch program. Six encoded sequences were considered to be candidate SCN R-genes (Figure 3). According to the conversed domain shared by all the R-genes, all of these six encoded sequences were identified to encode LRR-TM-PK protein [8]. It was notable that these encoded sequences were organized in a cluster on the chromosome 16 (inferred to be LG J). Four encoded sequences within this cluster of chromosome 16 were successfully amplified by RT-PCR and showed a high homology with other LRR-TM-PK type of resistance genes (data not shown). The results of semi-quantitative RT-PCR showed that Glyma16g30760.1 was only abundant in the SCN resistant cultivar 'L-10' but not in susceptible 'Heinong 37'. Further, the transcript abundance was higher in root than in leaf for 'L-10'. Therefore, the gene might play a role in the SCN resistance (Figure 4). Future analyses would be focused to determine the role in the resistance mechanism underlain by Glyma16g30760.1.

## Discussion

The SCN resistance line 'L-10' had a broad-spectrum resistant to SCN. There was great interest in transferring the SCN resistance of this cultivar into other cultivars. Traditional identification processes were time consuming and labor intensive. Increasing the selection intensity by marker-assisted selection of genotypes and subsequently more limited phenotypic selection will lead to improved the selection gains.

Segregation distortion had been reported repeatedly in soybean inbred line populations segregating for the soybean cyst nematode resistance locus *rhg1*, but the frequency of the SCN resistance allele was lower than expected [19]. In this study, QTL Qscn3-2 that was within the same region as the *rhg1 *locus was only detected in about one fourth of the population based on the flanking markers Satt275 and Satt309. The flanking markers Sat_350 and Satt244 for Qscn3-3 were only detected in nearly one third of the population. It was reported by Kopisch-Obuch et al. (2006) that the association between the resistance allele and reduced field emergence contributed to the segregation distortion at the SCN resistance loci [19]. In contrast, Webb et al. (1995) reported that either gametic or zygotic selection driven by a second locus on LG M that had to be inherited in phase were responsible [20]. In the present study, the RIL population performed a Skewness distribution due to non-typical quantitative trait of cyst nematode resistance. However, using the SAS normal logarithm conversion of the data resulted in normal distributions for these female index data, which could be used for QTL mapping.

Two major QTL (Qscn3-2 on LG G and Qscn3-3 on LG J) were identified to be significantly associated with the resistance to SCN race 3. The two QTL jointly explained about 40% of the total phenotypic variation. The QTL Qscn3-2 fell within a similar marker interval (Satt275-Satt309) previously reported associated with resistance to SCN race 3. This region was considered to be the *rhg1 *locus. The *rhg1 *locus had been shown to have an impact on SCN development in several resistance lines and provided resistance to many common SCN populations such as race 3 and 14. Many research groups have mapped the *rhg1 *locus on chromosome 18, to a location approximately 0.4 cM from Satt309 [21,22].

The interval on LG J contained both Qscn3-3 and Qscn14-4 (including *rhg5) *might underlie multi-races resistance to SCN in soybean [14]. In this study, no QTL was identified on the LG A2. The Rhg4 locus was considered to be another main QTL for SCN resistance, tightly linked to *I *locus [13]. Four alleles were known at *I*, the dominant allele inhibited seed coat pigmentation, leading to possession of a yellow seed coat, whereas the recessive i allele conferred a pigmented seed coat. Two other alleles, i^i ^and i^k^, gave rise to restricted pigmentation of the hilum and the saddle-shaped region, respectively [23]. In our work, there was no significant correlation between the color of seed coat (or hilum) and SCN resistance. This result indicated that the line 'L-10' had another locus for resistance to SCN race 3 independent of the Rhg4 locus. This locus may be similar to the LG J locus found in PI 88788 [7].

The SCN resistance alleles were previously associated with maturity, plant height, and lodging scores [19]. In contrast there were some *rhg1 *SCN resistance alleles from *G. soja *either had no effect or significantly (P < 0.05) enhanced yield compared with the susceptible alleles [24]. Here the correlation analysis suggested that some morphological traits such as plant height, color of pod coat, 100-seed weight and maturity were significantly associated with SCN resistance. Soybeans with tall stems, large seed size, late maturity and low protein content were correlated with SCN resistance for both race 3 and 14 (P < 0.05). The use of the 'L-10' SCN resistance loci would provide breeders with an alternative source of SCN resistance that was not associated with reduced yield.

Two SSR markers, Satt275 and Satt309 on MLG G, were tightly linked to the major QTL Qscn3-2. Sat_305 and Satt244 on MLG J were tightly linked to the QTL Qscn3-3 and Qscn14-4. The total phenotypic variation explained by Qscn3-2 and Qscn3-3 together was 39.0% and 41.3% for the year 2007 and 2008, respectively, and these four markers had potential use for marker-assisted selection of lines resistant to the SCN race 3. It was showed that nearly 80% of lines with the alleles of these four markers that were contributed by 'L-10' provided high resistance or completely immunity (FI = 0). As the *rhg1 *locus has been characterized as incompletely dominant, soybean lines heterozygous at this locus often allowed cyst formation at a rate intermediate between the genotype of homozygous resistant and homozygous susceptible [14,25]. Here some lines were still heterozygous at the *rhg1 *locus after MAS, suggesting a more accurate gene based selection was needed for the greater accuracy [8,26].

Qscn14-4 might underlie multi-races resistance in soybean. Kanazin et al. (1996) identified a gene cluster that contained eight RGAs on LG J in the similar region [27]. Further, six members of the RLK family were involved in one cluster on Chromosome J. Among these the RLK gene Glyma16g30760.1 was found to accumulate transcripts in the SCN resistance cultivar 'L-10' but not in 'Heinong 37' and the transcript abundance was higher in root than in leaf for 'L-10', suggesting that the gene might play a role in the SCN resistance to race 3 and race 14. Melito et al. (2010) suggested that the RLK at *rhg1 *was involved in root development but not nematode development as shown by others [25]. Unfortunately, the study of Melito et al. (2010) was flawed by the lack of an analysis of the target RLK protein abundance despite the available antibodies. Further studies of the RLKs at *rhg1 *and *rhg5 *will be needed for the clarification of the resistance mechanism and involvement of the candidate genes.

Glyma16g30760.1 was tightly linked to the QTL Qscn3-3 and Qscn14-4, indicating that this gene might be associated with both SCN race 3 and race 14 resistance. Therefore, a real-time PCR combined with molecular marker-based selection could be used for the future breeding of SCN resistant lines.

## Conclusions

The four main SSR markers, Satt275, Satt309, Sat_305 and Satt244, which were tightly linked to the major QTL for resistance to SCN on LG G and J, would be candidates for marker-assisted selection of lines resistant to the SCN race 3. Among the six RLK genes, Glyma16g30760.1 was found to accumulate transcripts in the SCN resistance cultivar 'L-10' but not in 'Heinong 37'. The transcript abundance was higher in root than in leaf for 'L-10', suggesting that the gene might play a role in the SCN resistance to race 3 and race 14.

## Methods

### Construction of population and isolation of SCN

The 140 F_5 _derived F_7 _recombinant inbred lines (RILs) were advanced by single-seed-descent from a cross between 'L-10' and 'Heinong 37'. 'L-10' was a soybean line broadly resistant to SCN developed by Northeast Agricultural University, Harbin, China and 'Heinong 37' was a SCN susceptible cultivar that had high yield in un-infested environments. The soil that contained SCN was collected from Yichun and Daqing regions of Heilongjiang province in China. The cysts were dislodged from roots on nested sieves (20 mesh over 60 mesh = 850-μm-pore sieve over 250-μm-pore sieve) with water spray. Each isolate was tested according to the method described using the original prescribed soybean differentials 'Pickett', 'Peking', PI 88788, and PI 90763. Lee 68 was used as the susceptible control [28]. Seeds of the soybean differentials were germinated in vermiculite and transplanted singly into 7.5-cm-d clay pots when cotyledons opened. Each cultivar or line was transplanted into five pots. Plants were inoculated 2 days later with 4,000 eggs and second-stage juveniles in a 10-ml suspension. All plants were grown in a greenhouse at 25-28°C. Thirty days after inoculation, the cysts and females were collected and counted at each location. The index as used by Golden et al. (1970) was calculated as follows: FI = (Number of cysts and females on detected plant)/(Average number of cysts and females on 'Lee68') × 100. FI > 10 was assigned "+" and FI < 10 was assigned "-" [29]. According to the classification standards described by Riggs and Schmitt (1988), the soil collected from Yichun contained Hg Type 0 (SCN race 3) ('Peking' (female index; FI 2%), 'PI 88788' (FI 3%), 'PI 90763' (FI 1%) and 'Pickett' (FI 3%) were used as the standard differentials). The soil collected from Daqing contained a Hg Type.3.- (SCN race 14) (The cultivar 'Hutcheson' was used as the susceptible control while Peking (FI 98%), PI 88788 (FI 3%) and PI 90763 (FI 101%) and Pickett (FI 68%) were used as the standard differentials to determine the race classification (HG Type)).

### Female index determination of population

Each line of the RILs was treated as above in the greenhouse at a temperature of 25-28°C and the record for the number of cysts and female of each line was as the same as above. Each recombinant inbred line (RIL) for each treatment contained five plants. A complete randomized design was used with three replicates. Each experiment was repeated twice.

### SSR marker detection

Genomic DNA was isolated from leaf samples according to the procedures described by Yu et al. (1999) [30]. PCR amplifications were performed in 96-well micro titer plates using the PTC-100TM thermal cycler. Oligo nucleotide sequences were contributed by USDA-ARS Plant Genome Program, Cornell University and Iowa State University (http://129.186.26.94/ssr.html). SSR PCR reactions were 20 μl containing 2 μl of genomic DNA (25 ng/μl), 1.5 μl MgCl_2 _(25 mM), 0.3 μl dNTP mixtures (10 mM), 2 μl 10 × PCR buffer, 2 μl SSR primer (2 uM), 0.2 μl Taq polymerase enzyme (10 units/μl), 12 μl ddH_2_0. The amplification temperature profiles were as follows; 2 min at 94°C, followed by 35 cycles of 30 sec at 94°C, 30 sec at 47°C, 30 sec at 72°C, then 5 min at 72°C. After the PCR reaction, PCR products were mixed with loading buffer (2.5 mg/ml bromophenol blue, 2.5 mg/ml diphenylamine blue, 10 mM EDTA, and 95% (v/v) formamide), denatured for 5 min at 94°C and then put on ice for 5 min. The PCR products were separated by electrophoresis on a 6% (w/v) denaturing polyacrylamide gel. Bands were directly detected after rapid silver staining [31].

### Data analyses

Linkages among the markers were analyzed with Mapmaker 3.0b, using the Kosambi mapping function. WinQTLCart2.1 was used to detect QTL between marker intervals by permutations (permutations 300; significance level <0.05)[32,33]. The genetic linkage map was constructed by Mapchart 2.1 [34].

### QTL projected and chromosome location

The locations of QTL were mapped on linkage groups and compared to those constructed in published studies. The markers that were polymorphic differed among studies. Therefore, in order to compare among QTL locations all relevant intervals were projected onto the GmComposite2003 map using the homothetic function [35,36]. The projection of QTL used the software BioMercator 2.1.

A local blastn search was performed to construct the gene map using a stringent BLAST search with a threshold E-values ≤ 1e-50. A total of 1015 SSR markers together with 709 RFLP markers were used as the query sequences that were obtained from NCBI (http://www.ncbi.nlm.nih.gov), and the soybean genome sequences Glyma 1.0 were used as the database file (http://www.phytozome.net). A script Blastm.pl that was compiled by Perl language was developed for the construction of a gene map based on the search results. The QTL projected on GmComposite2003 computed previously were then projected onto this gene map based on the ratio of genetic distance and physical distance (about 412 kbp per cM).

### Candidate gene detection

A list of genes within the consensus QTL on this gene to physical map was generated using the genome annotation data. The structural features of nucleotide binding sites (NBS), leucine rich repeats (LRR), Toll/Interleukin-1 receptors (TIR), and protein kinases (PK) were extracted from the InterProScan database and used as the domain list. Genes that contained those four domains were detected by using the hmmsearch program from the Hmmer software package [37]. For a further analysis, sequences with a similarity >90% were inferred to have the coiled-coil (CC) domain by the Pepcoil program, and the transmembrane domain was predicted by the TM-HMMer program [38,39].

### Transcript abundance analysis

In order to identify putative resistance genes for further analysis semi-quantitative RT-PCR was used to measure the transcript abundances. To quantify the relative amount of transcript accumulating for each of the genes and compare that between the resistance and susceptible cultivars, 1 μl of the standardized cDNA was used in a 50 μl PCR solution and cycled to within the predetermined linear amplification range. At 20 cycles before the end of the last cycle, primers for the gene encoding β-actin-1 were added at the end of the 72°C extension phase. This would generate cDNA PCR products encoded by the target genes and the β-actin-1 gene both within their linear amplification ranges. For confirmation a 2 μl aliquot was sampled after each two cycles after 20. The PCR products were then separated by agarose gel electrophoresis, visualized on the 2% (w/v) agarose and the band intensities compared [40].

## Authors' contributions

WC carried out the QTL projected and chromosome location participated in the gene clone and drafted the manuscript. LD carried out the female index determination. ZW participated in the female index determination. HH carried out the SSR marker detection. YH participated in the design of the study and performed the statistical analysis. HZ, MG and WL conceived of the study, and participated in its design and coordination and helped to draft the manuscript. All authors read and approved the final manuscript.
